# *Xanthomonas* bacteriophages: a review of their biology and biocontrol applications in agriculture

**DOI:** 10.1186/s12866-021-02351-7

**Published:** 2021-10-25

**Authors:** Ritah Nakayinga, Angela Makumi, Venansio Tumuhaise, William Tinzaara

**Affiliations:** 1 Department of Biological Sciences, Faculty of Science, Kyambogo University, P.O. Box 1, Kyambogo, Uganda; 2 Department of Animal and Human Health, General Biosciences, International Livestock Research Institute, P.O. Box 3070, Nairobi, 00100 Kenya; 3 Department of Agriculture, Faculty of Vocational Studies, Kyambogo University, P.O. Box 1, Kyambogo, Uganda

**Keywords:** Taxonomy, Distribution, Isolation source, Host range, Life cycle, Phage efficacy

## Abstract

Phytopathogenic bacteria are economically important because they affect crop yields and threaten the livelihoods of farmers worldwide. The genus *Xanthomonas* is particularly significant because it is associated with some plant diseases that cause tremendous loss in yields of globally essential crops. Current management practices are ineffective, unsustainable and harmful to natural ecosystems. Bacteriophage (phage) biocontrol for plant disease management has been of particular interest from the early nineteenth century to date. *Xanthomonas* phage research for plant disease management continues to demonstrate promising results under laboratory and field conditions. AgriPhage has developed phage products for the control of *Xanthomonas campestris* pv*. vesicatoria* and *Xanthomonas citri* subsp. *citri*. These are causative agents for tomato, pepper spot and speck disease as well as citrus canker disease.

Phage-mediated biocontrol is becoming a viable option because phages occur naturally and are safe for disease control and management. Thorough knowledge of biological characteristics of *Xanthomonas* phages is vital for developing effective biocontrol products. This review covers *Xanthomonas* phage research highlighting aspects of their ecology, biology and biocontrol applications.

**Supplementary Information:**

The online version contains supplementary material available at 10.1186/s12866-021-02351-7.

## Background

The genus *Xanthomonas*; is a well-studied group of plant-associated Gram-negative bacteria that belong to the family *Xanthomonadaceae* subclass Gammaproteobacteria [1]. An estimated 27 species is pathogenic to approximately 400 plants. These include but not limited to sugar cane, beans, cassava, cabbage, banana, citrus, tomatoes, pepper and rice [2]. The life cycle of *Xanthomonas* has two stages: epiphytic and endophytic [3]. The epiphytic stage starts once bacteria colonize the surfaces of a new plant using adhesion ligands such as bacteria surface polysaccharides [4], adhesion proteins [5], and type IV pili [6]. After colonization comes biofilm formation, which then protects the bacteria from environmental stress factors [7]. The endophytic stage is characterised by bacterial entry into plant tissue via lesions or stomata and eventual movement throughout the vascular system. The bacteria re-emerge onto the plant surfaces once their population reaches the threshold, transmission occurs to new hosts and the infection cycle repeats [3].

Although *Xanthomonas* species are well-studied, the genus remains responsible for many crop diseases that cause crop yield losses in economically important crops worldwide [2, 3].

The current management methods used to control *Xanthomonas*-associated diseases include de-budding, uprooting, burying and burning of infected plant tissues, sterilization of garden tools, and application of copper-based pesticides and antibiotics such as streptomycin [8–10]. The concerns raised about ineffective cultural practices, copper-based pesticide, antibiotic resistance problems, and environmental chemical contamination have piqued worldwide interest in *Xanthomonas* phage research and biocontrol application in agriculture.

Phages are viruses that infect and replicate in bacteria. Phage replication cycles include temperate and lytic pathways with the lytic pathway being the easier and more important pathway for employment in phage biocontrol. In the lytic pathway the phages bind to the surface of bacteria after which they inject their DNA and replicate inside the cell. This results in the production of phage progeny that lyse and kill the bacteria [11]. In the temperate pathway, once the phage has successfully bound and injected its DNA into the host, the phage may either stably integrate into the genome of the bacteria or enter into the lytic life cycle. Using temperate phages in phage biocontrol poses some disadvantages in that, once the phage inserts its genome into the bacterial DNA chromosome, the prophage is transmitted to daughter cells by horizontal gene transfer thereby providing undesirable genes that may aggravate bacterial disease, e.g. filamentous phage CTX Φ that encodes cholera toxin [12].

Historically, bacteriophage-based biocontrol specific for phytopathogen *Xanthomonas* dates back to the early nineteenth century, when a filtrate of decomposing cabbage stopped the spread of cabbage-rot disease caused by *Xanthomonas campestris* pv. *campestris,* [13]. Decades later, similar biocontrol success was reported with phage-containing lysates that inhibited bacterial spot disease in peach caused by *Xanthomonas campestris* pv. *pruni* [14, 15]. A number of phage applications have progressed from in-vitro experiments to field trials. These include studies on bacterial spot of tomato caused by *Xanthomonas campestris* pv. *vesicatoria* [16]; geranium bacterial blight caused by *Xanthomonas campestris* pv. *pelargonii* [17]; leaf blight of onion caused by *Xanthomonas axonopodis* pv. *allii* [18]; citrus canker and citrus bacterial spot caused by *Xanthomonas axonopodis* pv. *citri* and *Xanthomonas axonopodis* pv. *citrumelo* [19]; asiatic citrus canker caused by *Xanthomonas axonopodis* pv. *citri* [20] and *Xanthomonas citri* subsp. *citri* [21]; bacterial leaf blight of rice caused by *Xanthomonas oryzae* pv. *oryzae* [22, 23] and bacterial leaf blight of welsh onions caused by *Xanthomonas axonopodis* pv. *allii* [24]. Two *Xanthomonas* phage products manufactured by AgriPhage [25] have been shown to successfully control pathogens that cause tomato and pepper spot disease and citrus canker disease.

Owing to the growing interest in using *Xanthomonas* phages to control the genus *Xanthomonas*, this review emphasizes the taxonomy, ecology, biology and biocontrol applications.

## Main text

### Taxonomy of *Xanthomonas* phages

A total of 168 *Xanthomonas* phages described to date classify into orders: *Caudovirales* with 151 phages and *Tubulavirales* with 17 phages (Additional file 1). According to the International Committee on Taxonomy of Viruses (ICTV), *Caudovirales* contain 9 families [26] and *Xanthomonas* phages reported in literature or National Centre for Biotechnology Information (NCBI) database belong to 5 families namely: *Podoviridae*, *Siphoviridae*, *Myoviridae*, *Autographiviridae*, and *Herelleviridae* (Additional file 1). A total of 71 *Xanthomonas* phages belong to *Myoviridae*, 42 belong to *Podoviridae*, 34 belong to *Siphoviridae*, 17 belong to *Inoviridae*, 3 belong to *Autographiviridae* and 1 member to *Herelleviridae*. Order *Caudovirales* possess tubular tails that can be either long and contractile (*Myoviridae*), long and non-contractile (*Siphoviridae*), or short and non-contractile (*Podoviridae*, *Autographiviridae*) [26–28]. The capsids of *Caudovirales* are non-enveloped, exhibit icosahedral symmetry with a typical diameter of 45 and 170 nm and encapsidate linear double-stranded genomes. Their genome length is between 39,980 and 384,670 nucleotides, carries between 40 and 592 open reading frames and has a guanine-cytosine (GC) content between 40 and 66% (Additional file 1). On the other hand, *Tubulavirales* consist of one family; *Inoviridae*. They are filamentous virions that possess helical symmetry and non-enveloped capsid (Additional file 1). The inovirus genomes are small, circular, single-stranded DNA molecules that range between 6000 and 8500 nucleotides. The genome encodes between 9 and 14 open reading frames and has a GC content between 57 and 60% (Additional file 1).

### Ecology and host range

*Ecology: geographical distribution, environmental isolation source, host bacteria and plant disease*.

#### Geographical distribution

The geographical distribution of *Xanthomonas* phages spans parts of Asia, North America, South America, Europe, Zealandia and North Africa. The countries where the phages are isolated are summarized in Table 1. The *Xathomonas* phages are distributed across the world depending on the pathogen that is present in that part of the world.

**Table 1:** Country of isolation of *Xanthomonas* phages, their families and host strain/s they infect

Country of isolation	*Xanthomonas* phage/s	Family	Causative bacterium	Reference
China	Xop41	*Siphoviridae*	*X. oryzae* pv. *oryzae*	[29]
China	Xoo-sp1,Xoo-sp2, Xoo-sp3, Xoo-sp4, Xoo-sp5, Xoo-sp6, Xoo-sp7, Xoo-sp8, Xoo-sp9, Xoo-sp10, Xoo-sp11, Xoo-sp12, Xoo-sp13, Xoo-sp14, Xoo-sp15	*Siphoviridae*	*X. oryzae* pv. *oryzae*	[30]
China	X1, X2, X3, X4, X5	*Myoviridae*	*X. oryzae* pv. *oryzae*	[31]
China	Xoo-sp14	*Myoviridae*	*X. oryzae* pv. *oryzae*	[32]
China	Xoo-sp13	*Myoviridae*	*X. oryzae* pv. *oryzae*	[33]
China	Xf409	*Inoviridae*	*X. oryzae* pv. *oryzicola*	[34]
Taiwan	Xp10, Xp12, Xp20	*Siphoviridae*	*X. oryzae* pv. *oryzae*	[35]
Taiwan	ϕXc10	*Autographiviridae*	*X. citri* pv. *glycines*, *X. campestris* pv. *campestris*, *X. campestris* pv. *citri*	[36]
Korea	P8L, P27L, P30L, P59L, P73L	*Siphoviridae*	*X. oryzae* pv. *oryzae*	[22]
Korea	P4L, P4M, P6M, P6M1, P14M, P14M1, P18M, P23M1,P33M, P37L, P37M, P37M1, P41M, P43M, P45M, P47M, P50M, P53M, P54M, P57M, P58M, P60M, P61M, P62M, P66M, P68M, P70M, P71L, P72M	*Myoviridae*	*X. oryzae* pv. *oryzae*	[22]
Japan	XacN1	*Myoviridae*	*X. citri*	[37]
Viet Nam	Phage Xaa_vB_ϕ31	*Autographiviridae*	*X. euvesicatoria* pv*. allii* XaaBL11	[38]
Philippines	XPP1	*Myoviridae*	*X. oryzae* pv. *oryzae*	[39]
Philippines	XPP2	*Myoviridae*	*X. oryzae* pv. *oryzae*	[39]
Philippines	XPP3	*Myoviridae*	*X. oryzae* pv. *oryzae*	[39]
Philippines	XPP4	*Myoviridae*	*X. oryzae* pv. *oryzae*	[39]
Philippines	XPP6	*Myoviridae*	*X. oryzae* pv. *oryzae*	[39]
Philippines	XPP8	*Myoviridae*	*X. oryzae* pv. *oryzae*	[39]
Philippines	XPP9	*Myoviridae*	*X. oryzae* pv. *oryzae*	[39]
Philippines	XPV1	*Myoviridae*	*X. oryzae* pv. *oryzae*	[39]
Philippines	XPV2	*Myoviridae*	*X. oryzae* pv. *oryzae*	[39]
Philippines	XPV3	*Myoviridae*	*X. oryzae* pv. *oryzae*	[39]
India	φXOF1	*Siphoviridae*	*X. oryzae* pv. *oryzae*	[23]
India	φXOF2	*Siphoviridae*	*X. oryzae* pv. *oryzae*	[23]
India	φXOF3	*Siphoviridae*	*X. oryzae* pv. *oryzae*	[23]
India	φXOF4	*Siphoviridae*	*X. oryzae* pv. *oryzae*	[23]
India	φXOT1	*Siphoviridae*	*X. oryzae* pv. *oryzae*	[23]
India	φXOT2	*Siphoviridae*	*X. oryzae* pv. *oryzae*	[23]
India	φXOM1	*Siphoviridae*	*X. oryzae* pv. *oryzae*	[23]
India	φXOM2	*Siphoviridae*	*X. oryzae* pv. *oryzae*	[23]
India	Xcc9SH3	*Siphoviridae*	*X. campestris* pv. *campestris*	[40]
India	Xcc3SH, Xcc6SH3, Xcc7SH3, Xcc8SH3, Xcc9SH3, Xcc14SH3, JPS-xcc-3_P1, JPS-xcc-4_P1, JPS-xcc-7_P1, NBL-xcc-7_P1, NBL-xcc-4_P1, NBL-xcc-7_P1, NBL-xcc-3_P1, NBL-xcc-9_P1,NFS-xcc-9_P1, GRW-xcc-9_P1, NFS-xcc-9_P2, NBL-xcc-9_P2, GRW-xcc-10_P1, NFS-xcc-10_P1, NBL-xcc-10_P1, GRW-xcc-14_P1, NFS-xcc-14_P1, NBL-xcc-14_P1, GRW-xcc-17_P1, NFS-xcc-17_P1, NBL-xcc-17_P1, GRW-xcc-19_P1, NFS-xcc-19_P1, NBL-xcc-19_P1	n/a	*X. campestris* pv. *campestris*	[40]
India	Xap-1, Xap-2, Xap-3, Xap-4, Xap-5	n/a	*X. axonopodis* pv. *punicae*	[41]
USA	T7-like podophage Pagan	*Autographiviridae*	*Xanthomonas* sp., rice isolate ATCC PTA-13101	[42]
USA	Cf2	*Inoviridae*	*X. citri* pv. *citri*	[43]
USA	Phage River Rider	*Podoviridae*	*X. fragariae*	[44]
Mexico	Xaf13	*Inoviridae*	*X. vesicatoria*	[45]
Mexico	ϕXaf18	*Myoviridae*	*X. vesicatoria*	[46]
Brazil	XC2	*Myoviridae*	*X. campestris* pv. *campestris*	[47]
Chile	f30-Xaj	*Podoviridae*	*X. arboricola* pv. *juglandis*	[48]
Chile	f20-Xaj	*Podoviridae*	*X. arboricola* pv. *juglandis*	[48]
Russia	DB 1	*Siphoviridae*	*X. campestris* pv. *campestris*	[49]
Serbia	Kɸ1, Kɸ15	*Myoviridae*	*X. euvesicatoria*	[50]
Serbia	Kɸ1, Kɸ2, Kɸ3, Kɸ4, Kɸ5, Kɸ6, Kɸ7, Kɸ8, Kɸ9, Kɸ15	n/a	*X. euvesicatoria*	[50]
New Zealand	BP60C1–3, Bp10, Bp20, Bp22	*Myoviridae*	*X. campestris* pv. *juglandis*	[51]
New Zealand	P1, P2, P3, P4, P5, P6, P7, P8, P9, P10, P11, P12, P13, P14, P15, P16, P17, P18, P19, P20, P21, P22, P23, P24, P25, P26	*Siphoviridae*	*X. arboricola* pv. *juglandis*	[52]
France	Phage Olaya	*Podoviridae*	*X. albilineans* CFBP2523	[53]
France	Phage Bolivar	*Podoviridae*	*X. albilineans* CFBP2523	[54]
France	Phage Usaquen	*Podoviridae*	*X. albilineans* CFBP2523	[55]
France	Phage Alcala	*Podoviridae*	*X. albilineans* CFBP2523	[56]
France	Phage Fontebon	*Podoviridae*	*X. albilineans* CFBP2523	[57]
France	Phage Soumapaz	*Podoviridae*	*X. albilineans* CFBP2523	[58]
Belgium	FoX7	*Myoviridae*	*X. campestris* pv. *campestris* GBBC 1412	[59]
Belgium	FoX6	*Myoviridae*	*X. campestris* pv. *campestris* GBBC 1412	[60]
Belgium	FoX5	*Myoviridae*	*X. campestris* pv. *campestris* GBBC 1419	[61]
Belgium	FoX3	*Myoviridae*	*X. campestris* pv. *campestris* GBBC 1420	[62]
Belgium	FoX2	*Myoviridae*	*X. campestris* pv. *campestris* GBBC 1419	[63]
Belgium	FoX1	*Myoviridae*	*X. campestris* pv. *campestris* GBBC 1419	[64]
Belgium	FoX4	*Siphoviridae*	*X. campestris* pv. *campestris* GBBC 1412	[65]
Moldova	Phage PPDBI	*Podoviridae*	*X. campestris* pv. *campestris*	[49]
Egypt	Phage 1, Phage 2	n/a	*X. axonopodis*	[66]

*n/a* not available; *X Xanthomonas*; *pv* pathovar; *sp* species

#### Ecology: environmental isolation source, host bacteria and plant disease

The environmental isolation source of *Xanthomonas* phages as well as bacterial host and plant disease are summarized in Table 2. These viruses establish infection in *Xanthomonas* pathovars responsible for a range of plant diseases including but not limited to bacterial leaf blight, black rot, bacterial leaf spot and citrus canker (Table 2). The majority of *Xanthomonas* phages are isolated from infected plant phyllosphere and rhizosphere, while others are isolated from compost, sewage and water (irrigation, pond, freshwater lakes and rivers) (Table 2).

**Table 2:** Ecology of selected *Xanthomonas* phages: environmental source of isolation, host bacteria and plant disease

*Xanthomonas* phage/s	Environmental source	Host bacterium	Plant disease	Plant	Reference
Xop411	*Xoo* infected leaves	*X. oryzae* pv. *oryzae*	Bacterial leaf blight	Rice	[29]
Xp12	*Xoo* infected paddy water	*X. oryzae* pv. *oryzae*	Bacterial leaf blight	Rice	[67]
P4L, P4M, P6M, P6M1, P14M, P14M1, P18M, P23M1,P33M, P37L, P37M, P37M1, P41M, P43M, P45M, P47M, P50M, P53M, P54M, P57M, P58M, P60M, P61M, P62M, P66M, P68M, P70M, P71L, P72M, P8L, P27L, P30L, P59L, P73L	*Xoo* infected paddy water	*X. oryzae* pv. *oryzae*	Bacterial leaf blight	Rice	[22]
XPP1-XPP9, XPV1-XPV3	*Xoo* infected paddy water & soil	*X. oryzae* pv. *oryzae*	Bacterial leaf blight	Rice	[39]
X1, X2, X3, X4, X5	*Xoo* infected leaves	*X. oryzae* pv. *oryzae*	Bacterial leaf blight	Rice	[31]
φXOF1-φXOF4, φXOT1- φXOT2, φXOM1-φXOM2	*Xoo* infected leaves	*X. oryzae* pv. *oryzae*	Bacterial leaf blight	Rice	[23]
Xoo-sp1, Xoo-sp2, Xoo-sp3, Xoo-sp4, Xoo-sp5, Xoo-sp6, Xoo-sp7, Xoo-sp8, Xoo-sp9, Xoo-sp10, Xoo-sp11, Xoo-sp12, Xoo-sp13, Xoo-sp14, Xoo-sp15	*Xoo* infected paddy soil	*X. oryzae* pv. *oryzae*	Bacterial leaf blight	Rice	[30]
Xf	*Xoo* infected leaves	*X. oryzae* pv. *oryzae*	Bacterial leaf blight	Rice	[68]
Xcc3SH, Xcc6SH3, Xcc7SH3, Xcc8SH3, Xcc9SH3, Xcc14SH3, JPS-xcc-3_P1, JPS-xcc-4_P1, JPS-xcc-7_P1, NBL-xcc-7_P1, NBL-xcc-4_P1, NBL-xcc-7_P1, NBL-xcc-3_P1, NBL-xcc-9_P1,NFS-xcc-9_P1, GRW-xcc-9_P1, NFS-xcc-9_P2, NBL-xcc-9_P2, GRW-xcc-10_P1, NFS-xcc-10_P1, NBL-xcc-10_P1, GRW-xcc-14_P1, NFS-xcc-14_P1, NBL-xcc-14_P1, GRW-xcc-17_P1, NFS-xcc-17_P1, NBL-xcc-17_P1, GRW-xcc-19_P1, NFS-xcc-19_P1, NBL-xcc-19_P1	*Xcc* infected soil and leaves, river water	*X. campestris* pv. *campestris*	Black rot	Crucifers; cabbage, cauliflower, brasicca	[40]
Pg125	*Xcc* infected swede seed, compost & sewage	*X. campestris* pv. *campestris*	Black rot	Crucifers; turnip, cabbage, swede	[69]
Xcc φ1	*Xcc* infected soil	*X. campestris* pv. *campestris*	Black rot	Crucifers;broccoli, cabbage, cauliflower, radish	[70]
XTP1	*Xcc* infected soil	*X. campestris* pv. *campestris*	Black rot	Crucifers; cabbage	[71]
XcaP1	*Xcc* infected leaves	*X. campestris* pv. *campestris*	Black rot	Crucifers; cabbage	[72]
XC2	*Xcc* infected leaves	*X. campestris* pv. *campestris*	Black rot	Crucifer; cauliflower	[47]
DB 1	*Xcc* infected soil	*X. campestris* pv. *campestris*	Black rot	Crucifer, cabbage	[49]
XcuP3	*Xcu* infected fruit	*X. campestris* pv. *cucurbitae*	Bacterial leaf spot	Pumpkin	[72]
XcuP1	*Xcu* infected leaves	*X. campestris* pv. *cucurbitae*	Bacterial leaf spot	Zucchini	[72]
XholP1	*Xho* infected Leaves	*X. campestris* pv. *holcicola*	Bacterial leaf streak	Sorghum	[72]
Xp3-I	*Xp* infected soil	*X. pruni*	Bacterial leaf spot	Peach	[15]
Xp3-A.	*Xp* infected soil	*X. pruni*	Bacterial leaf spot	Peach	[15]
XprP1	*Xpr* infected stem	*X. campestris* pv*. pruni*	Bacterial leaf spot	Plum	[72]
XmaP1	*Xma* infected leaves	*X. campestris* pv. *malvacearum*	Bacterial blight	Cotton	[72]
XveP1	*Xve* infected leaves	*X. campestris* pv. *vesicatoria*	Bacterial leaf spot	Goosberry	[72]
Kɸ1, Kɸ2, Kɸ3, Kɸ4, Kɸ5, Kɸ6, Kɸ7, Kɸ8, Kɸ9, Kɸ15	*Xeu* infected leaves, stems, fruits, soil, seeds & irrigation water	*X. euvesicatoria*	Bacterial leaf spot	Pepper	[50]
Phages I to XX	*Xtr* infected grains	*X. trifolii*	Wheat disease	Wheat	[73]
X. phage 1 & X. phage 2	*Xax* infected leaves	*X. axonopodis*	Bacterial leaf spot	Pepper	[66]
Xap-1, Xap-2, Xap-3, Xap-4, Xap-5	Pond water	*X. axonopodis pv. punicae*	Bacterial leaf blight	Pomegranate	[41]
1, 20, 22, ΦPS, ΦSD, ΦSL, ΦRS, Φ56, Φ112, Pg60	Sewage, compost, *Xp* infected soil, seed & dry bean straw	*X. phaseoli*	Common blight of beans	Beans	[74]
Pg176, Pg177, Pg181,	*Xp* infected soil	*X. phaseoli*	Common blight of beans	Beans	[75]
*Xanthomonas* Siphophage Samson	Sewage	*X.* sp. strain ATCC PTA-13101	Bacterial leaf blight	Rice	[76]
*Xanthomonas* phage pagan	Fresh water	*X.* sp. strain ATCC PTA-13101	Bacterial leaf blight	Rice	[42]
*Xanthomonas* phage XacN1	*Xci* infected soil	*X. citri*	Asian citrus canker	Orange	[37]
BP60C_1–3_, Bp_10_, Bp_20_, Bp_22_	*Xcj* infected soil	*X. campestris* pv. *juglandis*	Walnut blight	Walnut	[51]
P1-P26	*Xaj* infected soil, leaves & fruit	*X. arboricola* pv. *juglandis*	Walnut blight	Walnut	[52]
XaF13	*Xve* infected soil	*X. vesicatoria*	Bacterial leaf spot	Pepper	[45]

*X*, *Xanthomonas*; pv, Pathovar; sp., species; *Xanthomonas oryzae* pv. *oryzae*; *Xcc*, *Xanthomonas campestris* pv. *campestris*; *Xcu*, *Xanthomonas campestris* pv. *cucurbitae*; *Xho*, *Xanthomonas campestris* pv. *holcicola*; *Xp*, *Xanthomonas pruni*; *Xpr*, *Xanthomonas campestris* pv. *pruni*; *Xma*, *Xanthomonas campestris* pv. *malvacearum*; *Xve*, *Xanthomonas campestris* pv. *vesicatoria*; *Xeu*, *Xanthomonas euvesicatoria*; *Xtr*, *Xanthomonas trifolii*; *Xax*, *Xanthomonas axonopodis*; *Xp*, *Xanthomonas phaseoli*; *Xci*, *Xanthomonas citri*; *Xcj*, *Xanthomonas campestris* pv. *juglandis*; *Xaj*, *Xanthomonas arboricola* pv. *juglandis*; *Xve*, *Xanthomonas vesicatoria*

#### Host range

Phages with a narrow host range infect one or few of the same bacteria strains, broad host range phages infect multiple strains of the same bacteria, and polyvalent phages infect several species or unrelated genera [77, 78]. A total of 148 *Xanthomonas* phages described in literature have a narrow, broad or polyvalent host range. Of these 52 have a narrow and 88 have a broad host range. The remaining 8 have a polyvalent host range. The lytic activity of phages with a narrow host range is between 13 and 57% while those with a broad range is between 60 and 100% (Table 3).

**Table 3:** Host range of *Xanthomonas* phages

Host range	Phage	Bacteria strain used	Number bacteria strains	Lysed bacteria strains	% lytic activity	Reference
Narrow	*X. vesicatoria* phage (chilli derived)	*X. vesicatoria*	8	4	50	[79]
Narrow	*X. vesicatoria* phage (datura derived)	*X. vesicatoria*	8	1	13	[79]
Narrow	XC2	*X. campestris* pv. *campestris*	10	5	50	[47]
Broad	Xoo-sp1	*X. oryzae* pv. *oryzae*	10	9	90	[30]
Broad	Xoo-sp2	*X. oryzae* pv. *oryzae*	10	9	90	[30]
Broad	Xoo-sp3	*X. oryzae* pv. *oryzae*	10	9	90	[30]
Broad	Xoo-sp4	*X. oryzae* pv. *oryzae*	10	9	90	[30]
Broad	Xoo-sp5	*X. oryzae* pv. *oryzae*	10	9	90	[30]
Broad	Xoo-sp6	*X. oryzae* pv. *oryzae*	10	9	90	[30]
Broad	Xoo-sp7	*X. oryzae* pv. *oryzae*	10	9	90	[30]
Broad	Xoo-sp8	*X. oryzae* pv. *oryzae*	10	9	90	[30]
Broad	Xoo-sp9	*X. oryzae* pv. *oryzae*	10	9	90	[30]
Broad	Xoo-sp10	*X. oryzae* pv. *oryzae*	10	9	90	[30]
zBroad	Xoo-sp11	*X. oryzae* pv. *oryzae*	10	9	90	[30]
Broad	Xoo-sp12	*X. oryzae* pv. *oryzae*	10	9	90	[30]
Broad	Xoo-sp13	*X. oryzae* pv. *oryzae*	10	9	90	[30]
Broad	Xoo-sp14	*X. oryzae* pv. *oryzae*	10	9	90	[30]
Broad	Xoo-sp15	*X. oryzae* pv. *oryzae*	10	9	90	[30]
Broad	Kϕ1	*X. euvesicatoria*	59	59	100	[50]
Broad	Kϕ2	*X. euvesicatoria*	59	59	100	[50]
Broad	Kϕ3	*X. euvesicatoria*	59	59	100	[50]
Broad	Kϕ4	*X. euvesicatoria*	59	59	100	[50]
Broad	Kϕ5	*X. euvesicatoria*	59	59	100	[50]
Broad	Kϕ6	*X. euvesicatoria*	59	59	100	[50]
Broad	Kϕ7	*X. euvesicatoria*	59	59	100	[50]
Broad	Kϕ8	*X. euvesicatoria*	59	59	100	[50]
Broad	Kϕ9	*X. euvesicatoria*	59	59	100	[50]
Broad	Kϕ15	*X. euvesicatoria*	59	47	80	[50]
Broad	Xma-P1	*X.* pv. *malvacearum*	8	8	100	[72]
Broad	Xho-P1	*X. campestris* pv. *holcicola*	4	4	100	[72]
Broad	Xpr-P1	*X. campestris* pv. *pruni*	6	6	100	[72]
Broad	OP_2_	*X. oryzae* pv. *oryzae*	82	78	95	[80]
Broad	OP_1*h*2_	*X. oryzae* pv. *oryzae*	82	75	91	[80]
Narrow	OP_1_	*X. oryzae* pv. *oryzae*	82	46	56	[80]
Narrow	OP_1*h*_	*X. oryzae* pv. *oryzae*	82	20	24	[80]
Broad	φXOF1	*X. oryzae* pv. *oryzae*	6	4	67	[23]
Broad	φXOF2	*X. oryzae* pv. *oryzae*	6	4	67	[23]
Broad	φXOF3	*X. oryzae* pv. *oryzae*	6	5	83	[23]
Broad	φXOF4	*X. oryzae* pv. *oryzae*	6	6	100	[23]
Narrow	φXOT1	*X. oryzae* pv. *oryzae*	6	3	50	[23]
Narrow	φXOT2	*X. oryzae* pv. *oryzae*	6	3	50	[23]
Narrow	φXOM1	*X. oryzae* pv. *oryzae*	6	3	50	[23]
Narrow	φXOM2	*X. oryzae* pv. *oryzae*	6	3	50	[23]
Broad	X1	*X. oryzae* pv. *oryzae*	23	15	65	[31]
Broad	X2	*X. oryzae* pv. *oryzae*	23	21	91	[31]
Broad	X3	*X. oryzae* pv. *oryzae*	23	22	96	[31]
Broad	X4	*X. oryzae* pv. *oryzae*	23	21	91	[31]
Broad	X5	*X. oryzae* pv. *oryzae*	23	14	61	[31]
Broad	P4L	*X. oryzae* pv. *oryzae*	47	33	70	[22]
Broad	P4M	*X. oryzae* pv. *oryzae*	47	46	98	[22]
Broad	P6M	*X. oryzae* pv. *oryzae*	47	47	100	[22]
Broad	P6M1	*X. oryzae* pv. *oryzae*	47	47	100	[22]
Broad	P8L	*X. oryzae* pv. *oryzae*	47	36	77	[22]
Broad	P14M	*X. oryzae* pv. *oryzae*	47	47	100	[22]
Broad	P14M1	*X. oryzae* pv. *oryzae*	47	47	100	[22]
Broad	P18M	*X. oryzae* pv. *oryzae*	47	47	100	[22]
Broad	P23M1	*X. oryzae* pv. *oryzae*	47	47	100	[22]
Broad	P27L	*X. oryzae* pv. *oryzae*	47	33	70	[22]
Broad	P30L	*X. oryzae* pv. *oryzae*	47	31	66	[22]
Broad	P33M	*X. oryzae* pv. *oryzae*	47	47	100	[22]
Broad	P37L	*X. oryzae* pv. *oryzae*	47	33	70	[22]
Broad	P37M	*X. oryzae* pv. *oryzae*	47	47	100	[22]
Broad	P37M1	*X. oryzae* pv. *oryzae*	47	46	98	[22]
Broad	P41M	*X. oryzae* pv. *oryzae*	47	47	100	[22]
Broad	P43M	*X. oryzae* pv. *oryzae*	47	47	100	[22]
Broad	P45M	*X. oryzae* pv. *oryzae*	47	33	70	[22]
Broad	P47M	*X. oryzae* pv. *oryzae*	47	47	100	[22]
Broad	P50M	*X. oryzae* pv. *oryzae*	47	47	100	[22]
Broad	P53M	*X. oryzae* pv. *oryzae*	47	47	100	[22]
Broad	P54M	*X. oryzae* pv. *oryzae*	47	47	100	[22]
Broad	P57M	*X. oryzae* pv. *oryzae*	47	47	100	[22]
Broad	P58M	*X. oryzae* pv. *oryzae*	47	47	100	[22]
Broad	P59L	*X. oryzae* pv. *oryzae*	47	31	66	[22]
Broad	P60M	*X. oryzae* pv. *oryzae*	47	28	60	[22]
Broad	P61M	*X. oryzae* pv. *oryzae*	47	47	100	[22]
Broad	P62M	*X. oryzae* pv. *oryzae*	47	47	100	[22]
Broad	P66M	*X. oryzae* pv. *oryzae*	47	46	98	[22]
Broad	P68M	*X. oryzae* pv. *oryzae*	47	47	100	[22]
Broad	P70M	*X. oryzae* pv. *oryzae*	47	47	100	[22]
Narrow	P71L	*X. oryzae* pv. *oryzae*	47	27	57	[22]
Broad	P72M	*X. oryzae* pv. *oryzae*	47	47	100	[22]
Broad	P73L	*X. oryzae* pv. *oryzae*	47	46	98	[22]
Narrow	Xcc3SH	*X. campestris* pv. *campestris*	17	6	35	[40]
Narrow	Xcc7SH	*X. campestris* pv. *campestris*	17	5	29	[40]
Narrow	Xcc6SH	*X. campestris* pv. *campestris*	17	7	41	[40]
Narrow	Xcc8SH	*X. campestris* pv. *campestris*	17	4	24	[40]
Narrow	Xcc9LK	*X. campestris* pv. *campestris*	17	5	29	[40]
Broad	Xcc9SH3	*X. campestris* pv. *campestris*	17	17	100	[40]
Narrow	Xcc14SH	*X. campestris* pv. *campestris*	17	7	41	[40]
Narrow	JPS-xcc-3_P1	*X. campestris* pv. *campestris*	17	6	35	[40]
Narrow	JPS-xcc-4_P1	*X. campestris* pv. *campestris*	17	6	35	[40]
Narrow	JPS-xcc-7_P1	*X. campestris* pv. *campestris*	17	6	35	[40]
Narrow	NBL-xcc-7_P1	*X. campestris* pv. *campestris*	17	6	35	[40]
Narrow	NBL-xcc-4_P1	*X. campestris* pv. *campestris*	17	4	24	[40]
Narrow	NBL-xcc-7_P1	*X. campestris* pv. *campestris*	17	5	29	[40]
Narrow	NBL-xcc-3_P1	*X. campestris* pv. *campestris*	17	3	18	[40]
Narrow	NBL-xcc-9_P1	*X. campestris* pv. *campestris*	17	8	47	[40]
Narrow	NFS-xcc-9_P1	*X. campestris* pv. *campestris*	17	6	35	[40]
Narrow	GRW-xcc-9_P1	*X. campestris* pv. *campestris*	17	3	18	[40]
Narrow	NFS-xcc-9_P2	*X. campestris* pv. *campestris*	17	5	29	[40]
Narrow	NBL-xcc-9_P2	*X. campestris* pv. *campestris*	17	7	41	[40]
Narrow	GRW-xcc-10_P1	*X. campestris* pv. *campestris*	17	7	41	[40]
Narrow	NFS-xcc-10_P1	*X. campestris* pv. *campestris*	17	3	18	[40]
Narrow	NBL-xcc-10_P1	*X. campestris* pv. *campestris*	17	5	29	[40]
Narrow	GRW-xcc-14_P1	*X. campestris* pv. *campestris*	17	8	47	[40]
Narrow	NFS-xcc-14_P1	*X. campestris* pv. *campestris*	17	12	71	[40]
Narrow	NBL-xcc-14_P1	*X. campestris* pv. *campestris*	17	7	41	[40]
Narrow	GRW-xcc-17_P1	*X. campestris* pv. *campestris*	17	9	53	[40]
Narrow	NFS-xcc-17_P1	*X. campestris* pv. *campestris*	17	3	18	[40]
Narrow	NBL-xcc-17_P1	*X. campestris* pv. *campestris*	17	5	29	[40]
Narrow	GRW-xcc-19_P1	*X. campestris* pv. *campestris*	17	8	47	[40]
Narrow	NFS-xcc-19_P1	*X. campestris* pv. *campestris*	17	12	71	[40]
Narrow	NBL-xcc-19_P1	*X. campestris* pv. *campestris*	17	7	41	[40]
Broad	Pg60	*X. phaseoli*	16	15	94	[69]
Broad	Pg176	*X. phaseoli*	16	14	88	[69]
Narrow	Pg177	*X. phaseoli*	16	7	44	[69]
Narrow	Pg181	*X. phaseoli*	16	9	56	[69]
Broad	P1	*X. arboricora* pv. *juglandis*	16	14	88	[52]
Broad	P2	*X. arboricora* pv. *juglandis*	16	13	81	[52]
Broad	P3	*X. arboricora* pv. *juglandis*	16	12	75	[52]
Broad	P4	*X. arboricora* pv. *juglandis*	16	14	88	[52]
Broad	P5	*X. arboricora* pv. *juglandis*	16	13	81	[52]
Broad	P6	*X. arboricora* pv. *juglandis*	16	14	88	[52]
Broad	P7	*X. arboricora* pv. *juglandis*	16	10	63	[52]
Broad	P8	*X. arboricora* pv. *juglandis*	16	12	75	[52]
Broad	P9	*X. arboricora* pv. *juglandis*	16	11	69	[52]
Broad	P10	*X. arboricora* pv. *juglandis*	16	12	75	[52]
Broad	P11	*X. arboricora* pv. *juglandis*	16	12	75	[52]
Broad	P12	*X. arboricora* pv. *juglandis*	16	11	69	[52]
Broad	P13	*X. arboricora* pv. *juglandis*	16	11	69	[52]
Broad	P14	*X. arboricora* pv. *juglandis*	16	14	88	[52]
Broad	P15	*X. arboricora* pv. *juglandis*	16	14	88	[52]
Broad	P16	*X. arboricora* pv. *juglandis*	16	12	75	[52]
Broad	P17	*X. arboricora* pv. *juglandis*	16	12	75	[52]
Broad	P18	*X. arboricora* pv. *juglandis*	16	14	88	[52]
Broad	P19	*X. arboricora* pv. *juglandis*	16	14	88	[52]
Broad	P20	*X. arboricora* pv. *juglandis*	16	14	88	[52]
Broad	P21	*X. arboricora* pv. *juglandis*	16	11	69	[52]
Broad	P22	*X. arboricora* pv. *juglandis*	16	12	75	[52]
Narrow	P23	*X. arboricora* pv. *juglandis*	16	5	31	[52]
Narrow	P24	*X. arboricora* pv. *juglandis*	16	5	31	[52]
Narrow	P25	*X. arboricora* pv. *juglandis*	16	7	44	[52]
Narrow	P26	*X. arboricora* pv. *juglandis*	16	5	31	[52]
Narrow	ϕ5A	*X. axonopodis* pv*. allii*	12	5	42	[24]
Narrow	ϕ5B	*X. axonopodis* pv*. allii*	12	5	42	[24]
Broad	ϕ6	*X. axonopodis* pv*. allii*	12	9	75	[24]
Narrow	ϕ7A	*X. axonopodis* pv*. allii*	12	7	58	[24]
Narrow	Φ7B	*X. axonopodis* pv*. allii*	12	7	58	[24]
Narrow	Φ14	*X. axonopodis* pv*. allii*	12	6	50	[24]
Broad	Φ16	*X. axonopodis* pv*. allii*	12	11	92	[24]
Broad	Φ17A	*X. axonopodis* pv*. allii*	12	11	92	[24]
Broad	Φ17B	*X. axonopodis* pv*. allii*	12	9	75	[24]
Broad	Φ31	*X. axonopodis* pv*. allii*	12	12	100	[24]
Polyvalent	Pg125	*Xanthomonas* strains	52	52	100	[69]
Polyvalent	Xcu-Pl	*X. campestris* pv. *cucurbitae, X. campestris* pv. *dieffembachiae*, *X. campestris* pv. *holcicola*	38	26	68	[72]
Polyvalent	Xcu-P3	*X. campestris* pv*. cucurbitae*, *X. campestris* pv. *holcicola*	38	17	45	[72]
Polyvalent	Xve-P1	*X. campestris* pv. *pruni*, *X. campestris* pv. *vesicatoria*	38	9	24	[72]
Polyvalent	Xca-P1	*X. campestris* pv. *campestris*, *X. campestris* pv. *pruni*	38	15	39	[72]
Polyvalent	Xhol-P1	*X. campestris* pv*. cucurbitae, X. campestris* pv. *holcicola*	38	15	39	[72]
Polyvalent	Xma-P1	*X. campestris* pv*. cucurbitae*, *X. campestris* pv. *malvacearum*	38	14	37	[72]
Polyvalent	Xpr-P1	*X. campestris* pv*. holcicola*, *X. campestris* pv. *pruni*	38	15	39	[72]

*X Xanthomonas*; *pv* pathovar

The polyvalent *Xathomonas* phage Pg125, is lytic to multiple strains from 25 species within the genus *Xanthomonas* [69]. Others in this category include phage Xcu-Pl, Xcu-P3, Xve-P1, and Xca-P1 which are lytic to *Xanthomonas campestris* pathovars (Table 3). The varied host ranges demonstrated by *Xanthomonas* phages imply that these lytic viruses can offer viable plant disease management alternatives. The high level of host specificity minimizes the risk of phage attack on beneficial bacteria [50].

### Biology: physiological parameters

#### Incubation temperature, storage temperature, storage media

##### Incubation temperature

*Xanthomonas* phages can maintain their viability over a wide incubation temperature range. For example, *Xanthomonas phaseoli* phages (1, 20, 22, ΦPS, ΦSD, ΦSL, ΦRS, Φ56, Φ112, Pg60) remain viable between 2 and 28 °C [74]; *Xanthomonas pruni* phages (Xp3-A and Xp3-I) and *Xanthomonas oryzae* phages (Xp12 and φXOF4) between 20 and 50 °C [15, 23, 81] and *Xanthomonas euvesicatoria* phages (Kϕ1- Kϕ 15) between 35 and 70 °C [50].

##### Storage temperature

The storage temperature of *Xanthomonas* phages differs between strains. The initial titer 4 × 10^7^ pfu/ml of phage Kϕ1, is maintained for 6 months when stored at + 4 °C in nutrient broth, compared to storage at + 20 °C where it declines to 2 × 10^7^ pfu/ml within the same period [82]. Similarly, the lytic activity of *Xanthomonas trifolii* phages is maintained for a month at + 4 °C in phosphate buffer, pH 7 [73]. On the contrary, *Xanthomonas arboricora* phages (P6, P11, P15, P16, P20) survive poorly at + 4 °C in double distilled water during a one-year storage period. The initial phage titer (1 × 10^8^ pfu/ml) drops drastically to 1 × 10^3^ pfu/ml. The same phages decline to 8 × 10^4^ pfu/ml when maintained at − 34 °C in the same media [52]. Therefore, *Xanthomonas* phages are maintained longer when stored at + 4 °C in nutrient broth. The appropriate storage conditions for different phages should be determined in order to ensure longevity of their effectiveness during storage and prior to biocontrol applications [83].

##### Storage media, ionic strength and pH

Phage viability is dependent on the storage media, ionic strength and pH and these have to be optimal to ensure phage longevity.

Different types of storage media have been investigated to understand their effects on phage viability. SM buffer is a mixture of sodium chloride (100 mM), magnesium sulphate (10 mM), tris-HCL (50 mM, pH 7.5) and gelatin (0.01%). In addition to SM buffer is nutrient broth, water/chloroform (H_2_O-CHCl_3_) and nutrient broth/chloroform (NB-CHCl_3_) combinations [52]. The initial phage titer (1 × 10^10^ pfu/ml) of *Xanthomonas arboricora* phages drops to 1 × 10^6^ pfu/ml in SM buffer and to 1 × 10^5^ pfu/ml in nutrient broth and water/chloroform during a one-year period at + 4 °C. In addition, phage titers decline further down to 1 × 10^4^ pfu/ml under nutrient/chloroform combination [52]. In other studies, nutrient broth and SM buffer are favorable storage media for phage viability at + 4 °C for long-term storage. For example, the initial titer, 8 × 10^10^ pfu/ml of phage Kϕ1 declines slightly to 8 × 10^9^ pfu/ml in nutrient broth and SM buffer at + 4 °C during a three-week storage period [82]. Further decline in phage titer of 3 × 10^9^ pfu/ml is detected in sterile tap water and 10 mM magnesium sulphate while in distilled water the titers sharply fall to 3 × 10^7^ pfu/ml at the same storage temperature and period [82]. Therefore, SM buffer is a better medium for phage survival than nutrient broth, tap water, magnesium sulphate, water/chloroform and nutrient broth/chloroform combinations [52]. The right storage media type will preserve the structural integrity of the phage and retain their infectivity during long-term storage [83].

The effect of ionic strength (salt concentration in liquid media) and pH on phage viability has been studied for a few *Xanthomonas* phages. Xp12 and Cf, lytic activity is maintained in distilled water or 0.1 M phosphate buffer, pH 7.0. However, the ability of these phages to lyse bacterial cells is prevented when they are stored in normal saline (0.9% sodium chloride) or 0.1 M citrate phosphate buffer, pH 7.0 [67, 84]. The optimal pH of *Xanthomonas* phages is between 5 and 11, with a number of phages being stable in acidic conditions such as pH 4 [23, 67, 82, 85].

##### Ultraviolet irradiation and chloroform resistance

The phyllosphere is a hostile environment and many factors such as ultraviolet (UV) irradiation prevent phage persistence and survivability [86]. As with all phages, *Xanthomonas* phages are inactivated by UV light. Formulations that increase phage survival consist of milk, corn and sucrose, minimizing UV-induced damages that result from the production of thymine dimers [82, 87, 88].

Chloroform treatment during isolation and enrichment process is used to release phage and kill host bacteria [89]. With the exception of Xf and Cf, many *Xanthomonas* phages are resistant to chloroform treatment because they lack a lipid envelope that surrounds the capsid. The organic solvent disrupts lipid membranes and inactivates the phage [23, 50, 52, 74, 82, 90]. The ability to resist chloroform denaturation makes non-enveloped *Xanthomonas* phages easy to isolate, culture and maintained for long-term storage [88].

### Biology: life cycle, replication parameters and molecular mechanisms

#### Life cycle

Generally, clear plaques on a bacterial lawn could suggest that phages may have lytic life cycles, while turbid plaques represent temperate life cycles [91]. *Xanthomonas* phages produce both lytic and turbid plaques (Table 4). The latter outcome is due to the absence of bacterial host lysis resulting from phage genome integration into host bacteria chromosomes, causing latent infection [27]. Genome integration is facilitated by host XerC/D recombinases that mediate site-specific recombination of the phage genome into a 15 base-pair *dif* locus of the bacterial genome [93, 98]. Unlike lytic phages, temperate phages are not suitable for use as biocontrol agents due to their ability to cause lysogenic conversion, induction of superinfection immunity and increased risk of horizontal gene transfer [83].

**Table 4:** Life cycle of *Xanthomonas* phages

Phage	Life cycle	Host bacteria	Reference
Cp1	Lytic	*X. axonopodis* pv. *citri*	[92]
Cp2	Lytic	*X. axonopodis* pv. *citri*	[92]
XP3-A	Lytic	*X. pruni*	[15]
XP3-I	Lytic	*X. pruni*	[15]
Kϕ1	Lytic	*X. euvesicatoria*	[50]
Kϕ8	Lytic	*X. euvesicatoria*	[50]
Kϕ15	Lytic	*X. euvesicatoria*	[50]
Kϕ1–9 and Kϕ15	Lytic	*X. euvesicatoria*	[50]
Xoo-sp2	Lytic	*X. oryzae* pv. *oryzae*	[30]
Xoo-sp1–15	Lytic	*X. oryzae* pv. *oryzae*	[30]
Xp12	Lytic	*X. oryzae* pv. *oryzae*	[81]
X1	Lytic	*X. oryzae* pv. *oryzae*	[31]
X2	Lytic	*X. oryzae* pv. *oryzae*	[31]
X3	Lytic	*X. oryzae* pv. *oryzae*	[31]
X4	Lytic	*X. oryzae* pv. *oryzae*	[31]
X5	Lytic	*X. oryzae* pv. *oryzae*	[31]
φXOF4,φXOF1,φXOF 2,φXOF3, φXOT1,φXOT2,φXOM1	Lytic	*X. oryzae* pv. *oryzae*	[23]
P4L, P4M, P6M, P6M1, P14M, P14M1, P18M, P23M1,P33M, P37L, P37M, P37M1, P41M, P43M, P45M, P47M, P50M, P53M, P54M, P57M, P58M, P60M, P61M, P62M, P66M, P68M, P70M, P71L, P72M, P8L, P27L, P30L, P59L, P73L	Lytic	*X. oryzae* pv. *oryzae*	[22]
XTP1	Lytic	*X. campestris* pv. *campestris*	[71]
XC2	Lytic	*X. campestris* pv. *campestris*	[47]
Xcc9SH3	Lytic	*X. campestris* pv. *campestris*	[40]
P125	Lytic	*Xanthomonas* sp.	[69]
Xcu-P1	Lytic/Temperate	*X. campestris* pv. *cucurbitae*	[72]
Xcu-P3	Lytic/Temperate	*X. campestris* pv. *cucurbitae*	[72]
XholP1	Lytic/Temperate	*X. campestris* pv. *holcicola*	[72]
XmaP1	Lytic/Temperate	*X. campestris* pv. *malvacearum*	[72]
XcaP1	Lytic/Temperate	*X. campestris* pv. *campestris*	[72]
XprP1	Lytic/Temperate	*X. campestris* pv. *pruni*	[72]
XveP1	Lytic/Temperate	*X. campestris* pv. *vesicatoria*	[72]
P1 - P26	Lytic	*X. arboricola* pv. *juglandis*	[74]
1, 20, 22, ΦPS, ΦSD, ΦSL, ΦRS, Φ56, Φ112, Pg60	Lytic	*X. phaseoli*	[74]
Cf16	Temperate	*X. campestris* pv. *citri*	[93]
Cf1t	Temperate	*X. campestris* pv. *citri*	[94]
Cf16v1	Temperate	*X. campestris* pv. *citri*	[90]
ϕLf	Temperate	*X. campestris* pv. *campestris*	[95]
Cf1c	Temperate	*X. campestris* pv. *citri*	[96]
XacF1	Temperate	*X. axonopodis* pv. *citri*	[20]
Xf109	Temperate	*X. oryzae pv. oryzae*	[97]
XaF13	Temperate	*X. vesicatoria*	[45]
Xf	Temperate/carrier state	*X. oryzae* pv*. oryzae*	[68]
Cf	Temperate/carrier state	*X. citri*	[84]
ϕL7	Lytic	*X. campestris* pv. *campestris*	[95]

*X Xanthomonas*; *pv* pathovar; *sp* species

During adsorption, *Xanthomonas* phages bind to different bacteria host cell surface receptors [99]. The adsorption of phage ΦL7 onto *Xanthomonas campestris* pv. *campestris* requires binding to a complex receptor consisting of lipopolysaccharide and a secondary protein on the outer membrane.

Other filamentous phages such as Cf use the host pili (pilR) to bind to *Xanthomonas campestris* pv. *citri* [94, 100]. The phage then penetrates using chaperon proteins such as, TonB, ExbB, and ExbD1 encoded by operon, *tonB–exbB–exbD1–exbD2* [101, 102]. The host bacteria are lysed by peptidoglycan glycohydrolase, which is located in the phage tail [103].

#### Replication parameters

The replication of phages is studied using the one-step growth experiment which measures the latent period and burst size of a phage on a specific bacterium. These are essential parameters in the description of phage properties. The latent period is the period between initial phage adsorption to a host cell to lysis and release of progeny viruses [91]. *Xanthomonas* phages have short latent periods ranging from 20 to 45 min to moderate periods, 60 to 90 min (Table 5). Very long latent periods ranging from 120 to 210 min occur for P125, Xoo-sp2, Xp12 (*Siphorividae*) and XTP (*Myoviridae*) (Table 5). The burst sizes range from 4.6 to 350 virions per infected cell (pfu/cell), with P125 showing the lowest burst size (4.6 pfu/cell) and Xoo-sp2 with the highest burst size (350 pfu/cell) (Table 5).

**Table 5:** Replication parameters of studied *Xanthomonas* phages

Phage	Host Bacterium	Family	Latent Period (Min)	Burst size (pfu/cell)	MOI	Phage Adsorption Temperature Time (min)	Reference
Cp1	*X. axonopodis* pv. *citri*	*Siphoviridae*	60	20	1	28 °C 10	[92]
Cp2	*X. axonopodis* pv. *citri*	*Podoviridae*	90	100	1	28 °C 10	[92]
P5	*X. axonopodis* pv. *citri*	n/a	40	60%	n/a	25 °C 20	[83]
Xp3-A	*X. pruni*	n/a	30–45	42–49	0.1	27 °C 20	[15]
Xp3-I	*X. pruni*	n/a	60–75	176–256	0.1	27 °C 20	[15]
Kϕ1	*X. euvesicatoria*	*Myoviridae*	20	75+/−4	0.1	27 °C 5	[50]
Kϕ8	*X. euvesicatoria*	*Myoviridae*	30	74+/−22	0.1	27 °C 5	[50]
Kϕ15	*X. euvesicatoria*	*Myoviridae*	30	70+/−11	0.1	27 °C 5	[50]
Xoo-sp2	*X. oryzae* pv. *oryzae*	*Siphoviridae*	180	350	0.1	28 °C 10	[30]
Xp12	*X. oryzae* pv. *oryzae*	*Siphoviridae*	140	35	0.1	28 °C -	[81]
X1	*X. oryzae* pv. *oryzae*	*Myoviridae*	20	88	10	30 °C 15	[31]
X2	*X. oryzae* pv. *oryzae*	*Myoviridae*	20	88	0.001	30 °C 15	[31]
X3	*X. oryzae* pv. *oryzae*	*Myoviridae*	40	50	0.01	30 °C 15	[31]
X4	*X. oryzae* pv. *oryzae*	*Myoviridae*	20	75	1	30 °C 15	[31]
X5	*X. oryzae* pv. *oryzae*	*Myoviridae*	20	100	1	30 °C 15	[31]
φXOF4	*X. oryzae* pv. *oryzae*	*Siphoviridae*	20–30	1.8 × 10^7^ pfu/ml	0.1	28 °C 10	[23]
XTP1	*X. campestris* pv. *campestris*	*Myoviridae*	120	30–35	1	30 °C 15	[71]
*X. phaseoli phage*	*X. phaseoli*	*Siphoviridae*	30–45	40	n/a	22 °C 25	[104]
P125	*Xanthomonas* sp.	*Siphoviridae*	210	4.6	n/a	27 °C 30	[69]

*X*, *Xanthomonas*; sp., species; (n/a) not available in literature; min, minutes; MOI, multiplicity of infection; pfu, plaque forming unit; %, percentage; ml, milimeters

The multiplicity of infection (MOI) of reported *Xanthomonas* phages lie between 0.001 to 1, with the lowest observed for phage X2 at 0.001, and highest for X4, X5 and XTP1 at 1 (Table 5). It has been reported that phages with short latent period and high burst size have more efficient replication cycles [105]. Also, the optimal temperature and incubation time are essential parameters during phage adsorption. These conditions range between 22 and 30 °C, while incubation times are between 5 and 30 min for *Xanthomonas* phages (Table 5).

#### Molecular mechanisms

Phage-bacterial infection induces molecular changes that include DNA methylation, phosphorylation and transcription. DNA methylation is well-studied in phage Xp12 [81]. Upon infection in *Xanthomonas oryzae* pv*. oryzae*, Xp12 induces biosynthesis of an unusual base, 5-methylcytosine, that replaces all cytosine residues in the DNA of Xp12 [81]. The rest of the bases; adenine, thymine, and guanine, remain unaltered [67, 81]. DNA methylation confers unique physical and chemical properties upon Xp12 DNA i.e., acquisition of a low buoyant density and high melting temperature, compared to typical DNA [106]. The Xp12 phage-infected bacterial cells produce an enzyme deoxycytidylate methyltransferase, that catalyzes the direct methylation of deoxycytidine monophosphate (dCMP) to 5-methylcytosine, in the presence of tetrahydrofolic acid [107, 108].

Modification of phosphorylation occurs during *Xanthomonas* phage infection. When Xp12 infects *Xanthomonas oryzae* pv*. oryzae*, phosphorylation of three proteins is induced. The phosphorylated proteins 28 kDa, 28.5 kDa and 45 kDa in size are present only on infected cells. This type of molecular modification is suggestive of the existence of a phage specific regulatory mechanism involved during phage infection [109].

Transcriptional modifications are initiated upon phage-bacterial infection. In phage Xp10, infecting *Xanthomonas oryzae* pv*. oryzae* displays complete loss of transcription activity due deactivation of host RNA polymerase resulting from dissociation of the δ subunit from the host core RNA polymerase [110]. Later studies show that Xp10 reverts the transcription process by encoding an anti-termination factor p7 that allows formation of RNA transcripts by host RNA polymerase [111].

### Biocontrol applications of *Xanthomonas* phages

This section explores several approaches where *Xanthomonas* phages are employed as biocontrol agents to manage *Xanthomonas* species in either greenhouse or field conditions. These methods have been successful at either inhibiting *Xanthomonas* growth or reducing disease severity. These include, but are not limited to use of monophages or cocktail treatments, phage mixtures with non-pathogenic or with pathogenic bacteria, phage combinations with antibiotics or plant inducers, UV- protectants and phage mutants [16, 21, 24, 30, 88, 112, 113].

To date, two *Xanthomonas* phage-based products are commercially available for the biocontrol of tomato, pepper spot and citrus canker [25]. The earliest evidence of *Xanthomonas* phage application was published in the early nineteenth century by Mallmann & Hemstreet [13], who determined that filtrate from decomposing cabbage applied to rotting cabbage inhibits the growth of *Xanthomonas campestris* pv. *campestris* in infected tissue. Since then, other forms of phage mixtures have been investigated.

Civerolo [114] applied crude lysates of lytic phage cocktail (Xp3-A and Xp3-I) on peach seedling foliage, 1–2 h before infection with *Xanthomonas pruni* under greenhouse conditions. Only 6–8% of leaves were infected, and the disease significantly reduced to 17–31% compared with 96% recorded on the water-treated control plants. In addition, application of either Xp3-A or Xp3-I mixed with *Xanthomonas pruni* and applied immediately before pathogen inoculation resulted in a 51–54% decrease of bacterial spot symptoms in peach seedlings under similar environmental settings. Therefore, the use of the phage cocktail significantly reduced disease severity better than single phage-pathogen mixture. This could be due to the synergy between the replication characteristics of both phages in the cocktail i.e. the latent period of Xp3-A and Xp3-I is 30–45 min and 60–75 min, whereas the burst size is 42–49 and 176–256 pfu/cell [114].

Some studies disagree with the evidence that supports the benefits provided by cocktail phage biocontrol of *Xanthomonas* associated diseases. In a recent study [24], spray application of a purified phage cocktail made up of three phages (ɸ16, ɸ17A, ɸ31) failed to inhibit the growth *Xanthomonas axonopodis* pv. *allii*, the causative agent of bacterial leaf blight of welsh onions. The cocktail treatment reduced infection of onion leaves to 43.3%, while a monophage phage treatment consisting ɸ31 reduced to 26.6% compared to the untreated, infected control leaves at 67.5% at 9 days after inoculation. Phage ɸ31, family *Autographiviridae*, had the broadest spectrum and lysed 12 out of 12 *Xanthomonas axonopodis* pv. *allii* strains, a trait that may contribute to its biological efficacy [24].

In another study [23], the phage φXOF4 inhibited the growth of *Xanthomonas oryzae* pv. *oryzae* that causes bacterial leaf blight. The seedlings treated with φXOF4 at a titer of 1 × 10^8^ pfu/ml showed no symptoms compared to 73% of the untreated group. Phage φXOF4, *Siphoviridae*, exhibited a broad host range where it lysed 6 out of 6 *Xanthomonas oryzae* pv. *oryzae* strains and had a short latent period between 20 and 30 min and a burst size that yields to the titer 1.8 × 10^7^ pfu/ml. There is preference for cocktail phages because of their ability to effectively control pathogenic strains and delay the emergence of resistant strains [115, 116]; however, studies [23, 24] support the evidence that monophage treatment can be effective at disease reduction or elimination.

Applications of premixed phage-pathogen suspensions are further demonstrated by Dong [30], who observed low treatment outcomes in rice plants treated with Xoo-sp2 and *Xanthomonas oryzae* pv. *oryzae* suspension. The average lesion length in treated plants was 13.31 ± 1.69 cm compared to two control groups treated in sterile water (20.83 ± 2.43 cm) or skimmed milk (19.29 ± 2.07 cm). Phage Xoo-sp2 (*Siphoviridae*) had a broad host range where it lysed 9 out of 10 *Xanthomonas oryzae* pv. *oryzae* strains and had a latent period of 180 min and burst size of 350 pfu/cell. Although the authors considered only Xoo-sp2 out of the 15 phages, a phage cocktail should have been considered to improve biocontrol efficacy since the remaining phages displayed equally a broad host range where they lysed 9 out of 10 of the same strains.

Alternative control approaches using non-pathogenic bacteria and phage suspensions are demonstrated by Nagai [112]. The combination of non-pathogenic *Xanthomonas* strain (npX, AXCB1201) and phage (pXS, XcpSFC211) was sprayed on broccoli plants before inoculation of *Xanthomonas campestris* pv. *campestris*. The npX-pXS mixture significantly reduced disease severity to 18.9% compared with 86.2% by pXS alone and 93.7% of water-treated control plants in greenhouse settings. Field trials showed a decrease in disease severity albeit lower than the results from the greenhouse experiments. The npX-pXS mixture reduced the symptoms by 74% compared to 98% of water treated control plants or 86% of copper treated plants [112].

Integration of *Xanthomonas* phages with antimicrobials or UV-protectants has been explored as a disease management option. Borah [117] found that the combination of phage (XMP-1) and antibiotic (streptomycin) suppressed leaf spot of mungbean caused by *Xanthomonas axonopodis* pv. *vignaeradiatae* to 4% compared with 68% of the untreated seedlings. Moreover, seed germination increased to 86% in comparison to 75% in the untreated group. Furthermore, Balogh [88] applied formulated phages on tomato plants infected with bacterial spot incited by *Xanthomonas campestris* pv. *vesicatoria*. The phages were mixed with either 0.5% pregelatinized cornflour (PCF), casecrete NH-400 with 0.25% PCF, or 0.75% powdered skim milk with 0.5% sucrose. Phage treatment improved plant yield by 62% (skim milk), 51% (Casecrete), and 30% (PCF) compared to unformulated phages at 1% in greenhouse experiments. Under field experiments, phage treatment increased plant yield by 18% (skim milk), 32% (casecrete) and 23% (PCF) compared to unformulated phages at 14%. Therefore, skim milk gave better results in greenhouse experiments while casecrete performed better in the field. Similarly, Tewfike and Shimaa [66] found that formulated phages in skim milk controlled better bacterial halo blight symptoms of pepper caused by *Xanthomonas axonopodis* than with corn flour by 20.5 and 18.3% in the greenhouse and 19.5 and 32.2% in field conditions.

Some studies have shown that unformulated phages can control better plant diseases. Balogh [19] applied unformulated phages to citrus leaves infected with asiatic citrus canker and recorded an average of 59% reduction in disease severity in five greenhouse experiments. The same phage mixture in skim milk was not effective at controlling disease under similar environmental settings. In nursery experiments, unformulated phage treatment also reduced disease, but was less effective than copper-mancozeb, a chemical bactericide. Moreover, mixing the unformulated phages with copper-mancozeb achieved comparable results to unformulated phages alone [19]. Therefore different field settings (greenhouse, open field and nursery beds) should be considered during biocontrol studies because there is a possibility that phage efficacy depends on the field settings.

Plant inducers successfully control plant diseases, and therefore form an integral part of disease management practices. The application of mixtures of phages in skim milk/sucrose with Acibenzolar-S-methyl (ASM), a plant inducer, decrease the bacterial spot of tomato caused by *Xanthomonas campestris* pv. *vesicatoria* under field conditions. The fruit yield of the formulated phage/ASM mixture was 67.9% compared to 60.8% of untreated control when applied twice biweekly in the first year [113]. Equally, Ibrahim [21] applied mixtures containing ASM and phages in skim milk/sucrose on citrus leaves for 4 days triweekly before inoculation of *Xanthomonas citri* subsp. *citri*, causative agent of asiatic citrus canker. Disease severity was reduced to 18.3% compared to 75.2% of the untreated control under greenhouse conditions. This observation agrees with results from field experiments where ASM/phages in skim milk/sucrose reduced disease to 12.5%, compared to 70.2% of the untreated control. When ASM was applied alone in the soil by drenching method, the disease was reduced to 38.2%, compared to 74.3% of the water-treated group after spraying 7 times triweekly before pathogen inoculation.

Mutated phages in formulations provide modest protection against plant disease compared with unformulated phages. The h-mutant phage mixtures (PMh; P4L, P43M, P23M1) in skim milk reduced bacterial blight disease of rice incited by *Xanthomonas oryzae* pv. *oryzae* to 18.1%, and wild type phage mixtures (PM; P4L, P43M, and P23M1) in the same formulation reduced the disease to 19.2%, compared to 39.1% of the untreated group. The mixtures were sprayed three times within an interval of 10 days. These tailed phages belong to the family *Myoviridae* and possess broad host range properties. Phage P4L lysed 33 out of 47, while P43M and P23M1 lysed 47 out of 47 *Xanthomonas oryzae* pv. *oryzae* strains [22]. Treatment with tecloftalam wettable powder, an agrochemical, demonstrated better results, with the disease symptoms reduced to 5% [22]. Therefore integration of tecloftalam wettable powder in plant protection could be a promising strategy for managing bacterial blight disease. On the contrary, agrochemicals have proved to be less effective than phages in controlling plant diseases. In a two-year greenhouse experiment, formulated phage DB1 in skim milk demonstrated improved black rot control by 71.1% while copper-based pesticide by 59.1%. Thus black rot caused by *Xanthomonas campestris* pv. *campestris* on cabbage seedlings can be successfully controlled by phage application [49].

Unformulated mutants reduce disease severity in infected plants. Flaherty [16] applied a mixture of host range mutant phages on tomato seedlings infected with *Xanthomonas campestris* pv. *vesicatoria* and symptoms of bacterial spot of tomato reduced to 0.9% compared to 40.5% of the untreated in the greenhouse. It increased the total weight of extra-large fruit by 14.9 and 24.2% in 1997 and 1998, respectively. Similarly, the severity of geranium bacterial blight declined when unformulated phage mutant mixtures were applied daily by foliar sprays on potted and seedling geraniums in greenhouse conditions [17].

Biofilm degradation is essential for the control of bacterial pathogenicity. The phage X3 causes 53% degradation of exopolysaccharide production and 43% biofilm degradation caused by *Xanthomonas oryzae* pv. *oryzae* that causes bacterial blight of rice [31]. When phage X3 was sprayed on rice plant foliage and seeds before pathogen inoculation, the plants improved by 83.1 and 95.4%. The phage X3 did not perform well when applied after pathogen inoculation, with results recorded between 28.9 and 73.9% [31]. Phage X3, family *Myoviridae*, had the broadest host range, lysed 22 out of the 23 *Xanthomonas oryzae* pv. *oryzae* strains tested and had the most extended latent period of 40 min with a burst size of 50 pfu/cell [31]. Likewise, infection of XacF1 (*Inoviridae*), a temperate phage, pathogenic to *Xanthomonas axonopodis* pv. *citri*, causing asiatic citrus canker, inhibits xanthan production, a component of extracellular polysaccharide that exacerbates the disease. The lesions on leaves sprayed with XacF1 reduced to 1 mm in width compared to 6.5 mm in untreated leaves. Therefore, the reduction in xanthan production caused by XacF1 phage reduces disease symptoms [20].

The frequency of phage spray and contact time on plant surfaces are factors investigated to improve the efficacy of phage applications. Lang [18] showed that multiple applications, i.e. biweekly or weekly applications of phages, effectively reduce symptoms of leaf blight of onion caused by *Xanthomonas axonopodis* pv. *allii* to 50%. Similar results were obtained when copper hydroxide-mancozeb was sprayed weekly on onion plants. Furthermore, biweekly application of Acibenzolar-S-methyl and phages reduced the disease by up to 50%. Hence, biweekly spray schedules are a promising strategy for sustainable control of leaf blight of onion.

Successful control of plant diseases is directly linked to the contact time of phages on plant surfaces. Gašić [82] successfully controlled bacterial pepper spot caused by *Xanthomonas euvesicatoria* by allowing a long contact time of phage Kϕ1 (*Myoviridae*) on plant leaves. The longest time of phage contact was 2 h before and 15 min after pathogen inoculation. This resulted in an average lesion number of 157, 213, and 189 compared to 332, 422, and 567 of the untreated control in three greenhouse experiments. The contact time experiments were further tested on copper hydroxide mixed with Kϕ1. At a contact time of 26 h before pathogen inoculation, a significant reduction in average lesion number was observed with scores of 63, 41, and 66 compared to 332, 422, and 567 of the untreated control. Thus longer contact time of phage Kϕ1 on plant surfaces allows effective control of pepper bacterial spot. There is a direct relationship between the timing of phage application and the efficacy of disease control. Evening applications of phage on foliage achieve better disease control since this period minimizes phage exposure to UV irradiation and extends phage longevity [88]. Phage Kϕ1 had the broadest host range where it lysed 59 out of 59 *Xanthomonas euvesicatoria* strains [50] and had a latent period and burst size of 20 min and 75 phage particles per infected cell respectively. Its multiplication and broad lytic abilities may contribute to its success at managing pepper bacterial spot.

The study of phage lysins as alternative biocontrol for *Xanthomonas* phytopathogens is rarely reported. One study has shown that phage lysozyme, Lys411, encoded by the genome of *Xanthomonas oryzae* phage, ϕXo411, can lyse *Xanthomonas* strains, making the protein a candidate with potential to control plant diseases caused by *Xanthomonas* [118].

One of the limitations faced by plant-based phage application is the hostile environment of the phyllosphere, where phages degrade rapidly due to desiccation or UV light. Phage formulations demonstrate protective benefits that enhance phage longevity and antibacterial activity [19, 88]; however, not all phages are effective in UV protectants [19]. Although, leaf surfaces of some plants do support phage multiplications, others do not; and this could potentially have adverse effects on the efficacy of a biocontrol product. Balogh [119] found that two *Xanthomonas perforans* phages (ɸXv3–21 and ɸXp06–02) multiplied and maintained populations on tomato leaf surface but did not achieve the same level of multiplication on grapefruit leaves. More research is needed to understand plant compounds involved and the mechanisms involved in this plant-phage interaction.

## Conclusion

Several *Xanthomonas* phages are evaluated for their potential as biocontrol agents against *Xanthomonas* species. So far, most of these belong to order *Caudovirales* and are lytic to a broad range of host strains. They are isolated from diverse ecosystems and distributed across the globe depending on the presence of the pathogen they infect. Their structural integrity and functionality in in vitro conditions is maintained under optimal growth and storage conditions. Pathogenesis of *Xanthomonas* phages in bacteria induce molecular alterations that may have regulatory functions important during their life cycle. Although few studies have focused on this aspect of biology, more research is needed to understand their life cycle.

From their first discovery in filtrates to applications as phage/pathogen suspensions, or in combination with other antimicrobials or with UV-protectants or as cocktail/monophage treatments, phages have proved to be promising alternatives to agrochemicals and antibiotics. They can reduce disease severity or inhibit bacteria growth in diverse field settings. So far, two *Xanthomonas* phage-based biocontrol products are commercially available for plant disease control. As the transition into commercial products continues, more studies are needed to tap into the many unexploited potentials of *Xanthomonas* phages for a range of *Xanthomonas* related plant diseases.

## Supplementary Information

**Additional file 1 **: **Table S1** Taxonomic classification, genomic properties and host bacteria of *Xanthomonas* phages. Description of data: *Xanthomonas* phages of order *Caudovirales* and *Tubulavirales*, their morphological and genomic properties and host bacteria.

## Data Availability

All data considered during this review is presented within the manuscript and in the additional supporting file.

## Abbreviations

ICTV: International Committee on Taxonomy of Viruses
nm: Nanometer
DNA: Deoxyribonucleic acid
GC: Guanine-Cytosine
ORF: Open Reading Frame
nts: Nucleotides
%: Percentage
pH: Potential of Hydrogen
NB: Nutrient broth
H_2_O: Water
CHCl_3_: Chloroform
M: Molarity
UV: Ultraviolet light
PFU: Plaque Forming Units
MOI: M*ultiplicity* of Infection
dCMP: Deoxycytidine monophosphate
kDa: Kilodalton
min: Minutes

## References

[1] Jun SR, Sims GE, Wu GA, Kim SH (2010). Whole-proteome phylogeny of prokaryotes by feature frequency profiles: an alignment-free method with optimal feature resolution. PNAS..

[2] Ryan RP, Vorhölter FJ, Potnis N, Jones JB, Van Sluys MA, Bogdanove AJ, Dow JM (2011). Pathogenomics of *Xanthomonas*: understanding bacterium-plant interactions. Nat Rev Microbiol.

[3] An SQ, Potnis N, Dow M, Vorhölter FJ, He YQ, Becker A, Teper D, Li Y, Wang N, Bleris L, Tang JL (2019). Mechanistic insights into host adaptation, virulence and epidemiology of the phytopathogen *Xanthomonas*. FEMS Microbiol Rev.

[4] Petrocelli S, Tondo ML, Daurelio LD, Orellano EG. Modifications of *Xanthomonas axonopodis* pv. *citri* lipopolysaccharide affect the basal response and the virulence process during citrus canker. PLoS One. 2012;7(7):e40051. 10.1371/journal.pone.0040051 PMC3391215 22792211

[5] Pradhan BB, Ranjan M, Chatterjee S (2012). XadM, a novel adhesin of *Xanthomonas oryzae* pv. *Oryzae*, exhibits similarity to Rhs family proteins and is required for optimum attachment, biofilm formation, and virulence. Mol. Plant Microbe Interact..

[6] Dunger G, Guzzo CR, Andrade MO, Jones JB, Farah CS (2014). *Xanthomonas citri* subsp. *citri* type IV pilus is required for twitching motility, biofilm development, and adherence. Mol. Plant Microbe Interact.

[7] Crossman L, Dow JM (2004). Biofilm formation and dispersal in *Xanthomonas campestris*. Microbes Infect.

[8] Biruma M, Pillay M, Tripathi L, Blomme G, Abele S, Mwangi M, Bandyopadhyay R, Muchunguzi P, Kassim S, Nyine M, Turyagyenda L, Eden-Green S (2007). Banana *Xanthomonas* wilt: a review of the disease, management strategies and future research directions. Afr J Biotechnol.

[9] Sundin GW, Wang N (2018). Antibiotic resistance in plant-pathogenic bacteria. Annu Rev Phytopathol.

[10] La Torre A, Iovino V, Caradonia F (2018). Copper in plant protection: current situation and prospects. Phytopathol Mediterr.

[11] Altamirano FLG, Barr JJ (2019). Phage therapy in the postantibiotic era. Clin Microbiol Rev.

[12] Doss J, Culbertson K, Hahn D, Camacho J, Barekzi N (2017). A review of phage therapy against bacterial pathogens of aquatic and terrestrial organisms. Viruses..

[13] Mallmann WL, Hemstreet C (1924). Isolation of an inhibitory substance from plants. J Agric Res.

[14] Civerolo EL, Kiel HL (1969). Inhibition of bacterial spot of peach foliage by *Xanthomonas pruni* bacteriophage. Phytopathol..

[15] Civerolo EL (1970). Comparative relationships between two *Xanthomonas pruni* bacteriophages and their bacterial host. Phytopathol..

[16] Flaherty JE, Jones JB, Harbaugh BK, Somodi GC, Jackson LE (2000). Control of bacterial spot on tomato in the greenhouse and field with H-mutant bacteriophages. HortScience..

[17] Flaherty JE, Harbaugh BK, Jones JB, Somodi GC, Jackson LE (2001). H-mutant bacteriophages as a potential biocontrol of bacterial blight of geranium. HortScience..

[18] Lang JM, Gent DH, Schwartz HF (2007). Management of *Xanthomonas* leaf blight of onion with bacteriophages and a plant activator. Plant Dis.

[19] Balogh B, Canteros BI, Stall RE, Jones JB (2008). Control of citrus canker and citrus bacterial spot with bacteriophages. Plant Dis.

[20] Ahmad AA, Askora A, Kawasaki T, Fujie M, Yamada T. The filamentous phage XacF1 causes loss of virulence in *Xanthomonas axonopodis* pv. *citri*, the causative agent of citrus canker disease. Front. Microbiol. 2014a;5:321. 10.3389/fmicb.2014.00321 PMC4076744 25071734

[21] Ibrahim YE, Saleh AA, Al-Saleh MA. Management of asiatic citrus canker under field conditions in Saudi Arabia using bacteriophages and acibenzolar-*S*-methyl. Plant Dis. 2017;101(5):761–5. 10.1094/PDIS-08-16-1213-RE 30678580

[22] Chae JC, Hung NB, Yu SM, Lee HK, Lee YH (2014). Diversity of bacteriophages infecting *Xanthomonas oryzae* pv. *Oryzae* in paddy fields and its potential to control bacterial leaf blight of rice. J Microbiol Biotechnol.

[23] Ranjani P, Gowthami Y, Gnanamanickam SS, Palani P (2018). Bacteriophages: a new weapon for the control of bacterial blight disease in rice caused by *Xanthomonas oryzae*. Microbiol Biotechnol Lett.

[24] Nga NTT, Tran TN, Holtappels D, Kim Ngan NL, Hao NP, Vallino M, Tien DTK, Khanh-Pham NH, Lavigne R, Kamei K, Wagemans J, Jones JB (2021). Phage biocontrol of bacterial leaf blight disease on welsh onion caused by *Xanthomonas axonopodis* pv *allii*. Antibiotics.

[25] OmniLytics. Sandy, Utah. 2021. https://www.omnilytics.com/ . Accessed 8 Mar 2021.

[26] Walker PJ, Siddell SG, Lefkowitz EJ, Mushegian AR, Adriaenssens EM, Dempsey DM (2020). Changes to virus taxonomy and the statutes ratified by the international committee on taxonomy of viruses. Arch Virol.

[27] Ackermann HW (1998). Tailed bacteriophages: the order *Caudovirales*. Adv Virus Res.

[28] King AMQ, Adams MJ, Carstens EB, Lefkowitz EJ (2012). Virus taxonomy. Ninth report of the international committee on taxonomy of viruses. 1st ed.

[29] Lee CN, Hu RM, Chow TY, Lin JW, Chen HY, Tseng YH, Weng SF (2007). Comparison of genomes of three *Xanthomonas oryzae* bacteriophages. BMC Genomics.

[30] Dong Z, Xing S, Liu J, Tang X, Ruan L, Sun M, Tong Y, Peng D (2018). Isolation and characterization of a novel phage Xoo-sp2 that infects *Xanthomonas oryzae* pv. *Oryzae*. J. Gen. Virol..

[31] Ogunyemi SO, Chen J, Zhang M, Wang L, Masum MMI, Yan C, An Q, Li B, Chen J (2019). Identification and characterization of five new OP2-related *Myoviridae* bacteriophages infecting different strains of *Xanthomonas oryzae* pv. *Oryzae*. J. Plant Pathol.

[32] National Center for Biotechnology Information. Bethesda Maryland. 2021. https://www.ncbi.nlm.nih.gov/nuccore/MT939492. Accessed 20 May 2021.

[33] National Center for Biotechnology Information. Bethesda Maryland. 2021. https://www.ncbi.nlm.nih.gov/nuccore/MN047793. Accessed 10 Mar 2021.

[34] National Center for Biotechnology Information. Bethesda Maryland. 2021. https://www.ncbi.nlm.nih.gov/nuccore/KY853667. Accessed 10 Mar 2021.

[35] Kuo TT, Huang TC, Wu RY, Yang CM (1967). Characterization of three bacteriophages of *Xanthomonas oryzae* (Uyeda et Ishiyama) Dowson. Bot Bull Acad Sinica.

[36] National Center for Biotechnology Information. Bethesda Maryland. 2021. https://www.ncbi.nlm.nih.gov/nuccore/MF375456. Accessed 10 Mar 2021.

[37] Yoshikawa G, Askora A, Blanc-Mathieu R, Kawasaki T, Li Y, Nakano M, Ogata H, Yamada T (2018). *Xanthomonas citri* jumbo phage XacN1 exhibits a wide host range and high complement of tRNA genes. Sci Rep.

[38] National Center for Biotechnology Information. Bethesda Maryland. 2021. https://www.ncbi.nlm.nih.gov/nuccore/MT951568. Accessed 20 May 2021.

[39] Kovács T, Molnár J, Varga I, Nagy IK, Valappil SK, Papp S, Vera Cruz CM, Oliva R, Vizi T, Schneider G, Rákhely G (2019). Complete Ggenome Ssequences of 10 *Xanthomonas oryzae* pv. *Oryzae* bacteriophages. Microbiol. Resour. Announc..

[40] Renu BMS, Singh UB (2017). Sahu U, Nagrale DT, Sahu PK. Characterization of lytic bacteriophage XCC9SH3 infecting *Xanthomonas campestris* pv. *Campestris*. J. Plant Pathol.

[41] Harshitha KN, Manoranjitham SK, Somasundaram E, Rajendran L, Karthikeyan G (2018). Characterization of *Xanthomonas axonopodis* pv. *punicae* (Hingorani and Singh) Vauterin et al. and isolation of Xap specific bacteriophage. Madras Agric. J.

[42] Russo M, Le T, Moreland R, Gonzalez CF, Liu M, Ramsey J (2019). Complete genome sequence of *Xanthomonas* phage pagan. Microbiol. Resour. Announc..

[43] National Center for Biotechnology Information. Bethesda Maryland. 2021. https://www.ncbi.nlm.nih.gov/nuccore/MK512531. Accessed 10 Mar 2021.

[44] Miller M, Deiulio A, Holland C, Douthitt C, McMahon J, Wiersma-Koch H, Turechek WW, D'Elia T (2020). Complete genome sequence of *Xanthomonas* phage river rider, a novel N4-like bacteriophage that infects the strawberry pathogen *Xanthomonas fragariae*. Arch Virol.

[45] Solis-Sanchez A, Quinones-Aguilar E, Vega-Arreguin J, Fraire-Velazquez S, Rincon-Enriquez G (2020). Complete genome sequence of XaF13 a novel bacteriophage of *Xanthomonas vesicatoria* from Mexico. Microbiol Resour Announc.

[46] National Center for Biotechnology Information. Bethesda Maryland. 2021. https://www.ncbi.nlm.nih.gov/nuccore/MN461279. Accessed 10 Mar 2021.

[47] da Silva FP, Xavier AD, Bruckner FP, de Rezende RR, Vidigal PMP, Alfenas-Zerbini P (2019). Biological and molecular characterization of a bacteriophage infecting *Xanthomonas campestris* pv. *Campestris*, isolated from brassica fields. Arch. Virol..

[48] Retamales J, Vasquez I, Santos L, Segovia C, Ayala M, Alvarado R, Nunez P, Santander J (2016). Complete genome sequences of lytic bacteriophages of *Xanthomonas arboricola* pv. *Juglandis*. Genome Announc.

[49] Orynbayev AT, Dzhalilov FSU, Ignatov AN (2020). Improved efficacy of formulated bacteriophage in control of black rot caused by *Xanthomonas campestris* pv. *Campestris* on cabbage seedlings. Arch. Phytopathol. Plant Prot.

[50] Gašić K, Ivanović MM, Ignjatov M, Calić A, Obradović A (2011). Isolation and characterization of *Xanthomonas euvesicatoria* bacteriophages. J Plant Pathol.

[51] McNeil DL, Romero S, Kandula J, Stark C, Stewart A, Larsen S (2001). Bacteriophages as a potential biocontrol agent against walnut blight (*Xanthomonas campestris* pv. *Juglandis*). N. Z. Plant Prot.

[52] Romero-Suarez S, Jordan B, Heinemann JA (2012). Isolation and characterization of bacteriophages infecting *Xanthomonas arboricola* pv. *Juglandis*, the causal agent of walnut blight disease. World J Microbiol Biotechnol.

[53] National Center for Biotechnology Information. Bethesda Maryland. 2021. https://www.ncbi.nlm.nih.gov/nuccore/MW802488. Accessed 20 May 2021.

[54] National Center for Biotechnology Information. Bethesda Maryland. 2021. https://www.ncbi.nlm.nih.gov/nuccore/MW822535. Accessed 20 May 2021.

[55] National Center for Biotechnology Information. Bethesda Maryland. 2021. https://www.ncbi.nlm.nih.gov/nuccore/MW822536. Accessed 20 May 2021.

[56] National Center for Biotechnology Information. Bethesda Maryland. 2021. https://www.ncbi.nlm.nih.gov/nuccore/MW822537. Accessed 20 May 2021.

[57] National Center for Biotechnology Information. Bethesda Maryland. 2021. https://www.ncbi.nlm.nih.gov/nuccore/MW822538. Accessed 20 May 2021.

[58] National Center for Biotechnology Information. Bethesda Maryland. 2021. https://www.ncbi.nlm.nih.gov/nuccore/MW822539. Accessed 20 May 2021.

[59] National Center for Biotechnology Information. Bethesda Maryland. 2021. https://www.ncbi.nlm.nih.gov/nuccore/MT161387. Accessed 20 May 2021.

[60] National Center for Biotechnology Information. Bethesda Maryland. 2021. https://www.ncbi.nlm.nih.gov/nuccore/MT161386. Accessed 20 May 2021.

[61] National Center for Biotechnology Information. Bethesda Maryland. 2021. https://www.ncbi.nlm.nih.gov/nuccore/MT161384. Accessed 20 May 2021.

[62] National Center for Biotechnology Information. Bethesda Maryland. 2021. https://www.ncbi.nlm.nih.gov/nuccore/MT161383. Accessed 20 May 2021.

[63] National Center for Biotechnology Information. Bethesda Maryland. 2021. https://www.ncbi.nlm.nih.gov/nuccore/MT161382. Accessed 20 May 2021.

[64] National Center for Biotechnology Information. Bethesda Maryland. 2021. https://www.ncbi.nlm.nih.gov/nuccore/MT161381. Accessed 20 May 2021.

[65] National Center for Biotechnology Information. Bethesda Maryland. 2021. https://www.ncbi.nlm.nih.gov/nuccore/MT161385. Accessed 20 May 2021.

[66] Tewfike TA, Shimaa MD (2015). Biocontrol of *Xanthomonas axonopodis* causing bacterial spot by application of formulated phage. Ann Agric Sci Moshtohor.

[67] Kuo TT, Huang TC, Wu RY, Chen CP (1968). Phage Xp12 of *Xanthomonas oryzae* (Uyeda et Ishiyama) Dowson. Can J Microbiol.

[68] Kuo TT, Huang TC, Chow TY (1969). A filamentous bacteriophage from *Xanthomonas oryzae*. Virology..

[69] Sutton MD, Katznelson H, Quadling C (1958). A bacteriophage that attacks numerous phytopathogenic *Xanthomonas* species. Can J Microbiol.

[70] Papaianni M, Cuomo P, Fulgione A, Albanese D, Gallo M, Paris D, Motta A, Iannelli D, Capparelli R (2020). Bacteriophages promote metabolic changes in bacteria biofilm. Microorganisms..

[71] Weiss BD, Capage MA, Kessel M, Benson SA (1994). Isolation and characterization of a generalized transducing phage for *Xanthomonas campestris* pv. *Campestris*. J. Bacteriol..

[72] Alippi AM (1989). Host range and particle morphology of some bacteriophages affecting pathovars of *Xanthomonas campestris*. Microbiologia..

[73] James N, Roslycky EB (1956). Specificity of bacterial virus for *Xanthomonas trifolii*. Can J Microbiol.

[74] Vidaver AK, Schuster ML (1969). Characterization of *Xanthomonas phaseoli* bacteriophages. J Virol.

[75] Sutton MD, Wallen VR (1967). Phage types of *Xanthomonas phaseoli* isolated from beans. Can J Bot.

[76] Clark S, Le T, Moreland R, Liu M, Gonzalez CF, Gill JJ, Ramsey J (2019). Complete genome sequence of *Xanthomonas* siphophage Samson. Microbiol. Resour. Announc..

[77] Ackermann HW, DuBow MS. "Phage multiplication". In: viruses of prokaryotes: general properties of bacteriophages, Ackermann HW, DuBow MS, editors. BocaRaton, Florida: CRC Press, Inc; 1987.p.49–85.

[78] Weinbauer MG (2004). Ecology of prokaryotic viruses. FEMS Microbiol Rev.

[79] Mathew J, Patel PN (1979). Host specific bacteriophages of *Xanthomonas vesicatoria*. J Phytopathol.

[80] Wakimoto S (1960). Classification of strains of *Xanthomonas oryzae* on the basis of their susceptibility against bacteriophages. J J Phytopathol.

[81] Kuo TT, Huang TC, Teng MH (1968). 5-Methylcytosine replacing cytosine in the deoxyribonucleic acid of a bacteriophage for *Xanthomonas oryzae*. J Mol Biol.

[82] Gašić K, Kuzmanović N, Ivanović M, Prokić A, Šević M, Obradović A (2018). Complete genome of the *Xanthomonas euvesicatoria* specific bacteriophage KΦ1, its survival and potential in control of pepper bacterial spot. Front Microbiol.

[83] Gill J, Hyman P (2010). Phage choice, isolation, and preparation for phage therapy. Curr Pharm Biotechnol.

[84] Dai H, Chiang KS, Kuo TT (1980). Characterization of a new filamentous phage Cf from *Xanthomonas citri*. J Gen Virol.

[85] Myung IS, Nam KW, Cho Y (2002). Cultural characteristics of *Xanthomonas axonopodis* pv. *Citri* bacteriophages CP1 from Korea. Plant Pathol. J..

[86] Iriarte FB, Balogh B, Momol MT, Smith LM, Wilson M, Jones JB (2007). Factors affecting survival of bacteriophage on tomato leaf surfaces. Appl Environ Microbiol.

[87] Wommack KE, Hill RT, Muller TA, Colwell RR (1996). Effects of sunlight on bacteriophage viability and structure. Appl Environ Microbiol.

[88] Balogh B, Jones JB, Momol MT, Olson SM, Obradovic A, King P, Jackson LE (2003). Improved efficacy of newly formulated bacteriophages for management of bacterial spot on tomato. Plant Dis.

[89] Schisler DA, Slininger PJ (1997). Microbial selection strategies that enhance the likelihood of developing commercial biological control products. J Ind Microbiol Biotechnol.

[90] Dai I, Chow TY, Liao HJ, Chen ZY, Chiang KS (1988). Nucleotide sequences involved in the neolysogenic insertion of filamentous phage Cf16-v1 into the *Xanthomonas campestris* pv.*Citri* chromosome. Virology..

[91] Clokie MRJ, Kropinski A. Bacteriophages: Methods and Protocols. Volume 1: Isolation, characterization, and interactions. 1^st^ ed. USA: Humana press, Springer Protocols;2009.

[92] Ahmad AA, Ogawa M, Kawasaki T, Fujie M, Yamada T (2014). Characterization of bacteriophages cp1 and cp2, the strain-typing agents for *Xanthomonas axonopodis* pv. *Citri*. Appl. Environ. Microbiol..

[93] Dai H, Tsay SH, Kuo TT, Lin YH, Wu WC. Neolysogenization of *Xanthomonas campestris* pv. *citri* infected with filamentous phage Cf16. Virology. 1987;156:313–320. 10.1016/0042-6822(87)90411-9 18644554

[94] Kuo TT, Lin YH, Huang CM, Chang SF, Dai H, Feng TY (1987). The lysogenic cycle of the filamentous phage Cflt from *Xanthomonas campestris* pv. *Citri*. Virology..

[95] Lee CN, Lin JW, Weng SF, Tseng YH (2009). Genomic characterization of the intron-containing T7-like phage phiL7 of *Xanthomonas campestris*. Appl Environ Microbiol.

[96] Kuo TT, Tan MS, Su MT, Yang MK (1991). Complete nucleotide sequence of filamentous phage Cf1c from *Xanthomonas campestris* pv. *citri*. Nucleic Acids Res.

[97] Yeh TY (2017). Complete nucleotide sequence of a new filamentous phage, Xf109, which integrates its genome into the chromosomal DNA of *Xanthomonas oryzae*. Arch Virol.

[98] Shieh GJ, Lin CH, Kuo JL, Kuo TT (1995). Characterization of an open reading frame involved in site-specific integration of filamentous phage Cflt from *Xanthomonas campestris* pv. *Citri*. Gene..

[99] Burmeister AR, Abedon ST, Turner PE. Bacteriophage ecology. Encycl Microbiol. 2019. 10.1016/B978-0-12-809633-8.90677-0.

[100] Yang YC, Chou CP, Kuo TT, Lin SH, Yang MK (2004). PilR enhances the sensitivity of *Xanthomonas axonopodis* pv. *Citri* to the infection of filamentous bacteriophage Cf. Curr. Microbiol..

[101] Hung CH, Wu HC, Tseng YH (2002). Mutation in the *Xanthomonas campestris* xanA gene required for synthesis of xanthan and lipopolysaccharide drastically reduces the efficiency of bacteriophage ΦL7 adsorption. Biochem Biophys Res Commun.

[102] Hung CH, Yang CF, Yang CY, Tseng YH (2003). Involvement of tonB-exbBD1D2 operon in infection of *Xanthomonas campestris* phage φL7. Biochem Biophys Res Commun.

[103] Weng SF, Fu YC, Lin JW, Tseng TT (2018). Identification of a broad-spectrum peptidoglycan hydrolase associated with the particle of *Xanthomonas oryzae*. Phage Xop411. J. Mol. Microbiol. Biotechnol..

[104] Katznelson H, Sutton MD, Bayley ST (1954). The use of bacteriophage of *Xanthomonas phaseoli* in detecting infection in beans, with observation on its growth and morphology. Can J Microbiol.

[105] Okabe N, Goto M (1963). Bacteriophages of plant pathogens. Annu Rev Phytopathol.

[106] Ehrlich M, Ehrlich K, Mayo JA (1975). Unusual properties of the DNA from *Xanthomonas* phage XP-12 in which 5-methylcytosine completely replaces cytosine. Biochim Biophys Acta.

[107] Kuo TT, Tu J (1976). Enzymatic synthesis of deoxy-5-methyl-cytidylic acid replacing deoxycytidylic acid in *Xanthomonas oryzae* phage Xp12 DNA. Nature..

[108] Kuo TT, Chow TY, Lin YT (1982). A new thymidylate biosynthesis in *Xanthomonas oryzae* infected by phage Xp12. Virology..

[109] Cheng CM, Tu J, Yang CC, Kuo TT (1994). Specific protein phosphorylation induced in *Xanthomonas campestris* pv. *Oryzae* by bacteriophage Xp12. Arch. Microbiol..

[110] Lin S-H, Liu J-S, Yang B-C, Kuo T-T (1998). Disassociation of sigma subunit from RNA polymerase of *Xanthomonas oryzae* pv. *Oryzae* by phage Xp10 infection. FEMS. Microbiol. Lett..

[111] Zenkin N, Severinov K, Yuzenkova Y (2015). Bacteriophage Xp10 anti-termination factor p7 induces forward translocation by host RNA polymerase. Nucleic Acids Res.

[112] Nagai H, Miyake N, Kato S, Maekawa D, Inoue Y, Takikawa Y (2017). Improved control of black rot of broccoli caused by *Xanthomonas campestris* pv. *Campestris* using a bacteriophage and a nonpathogenic *Xanthomonas* sp. strain. J. Gen. Plant Pathol.

[113] Obradovic A, Jones JB, Momol MT, Balogh B, Olson SM (2004). Management of tomato bacterial spot in the field by foliar applications of bacteriophages and SAR inducers. Plant Dis.

[114] Civerolo EL (1973). Relationship of *Xanthomonas pruni* bacteriophages to bacterial spot disease in Prunus. Phytopathol..

[115] Merabishvili M, Pirnay JP, De Vos D (1693). Guidelines to compose an ideal bacteriophage cocktail Methods. Mol Biol.

[116] Chan BK, Abedon ST, Loc-carrillo C. Phage cocktails and the future of phage therapy. Future Microbiol. 2013:769–83. 10.2217/fmb.13.47 23701332

[117] Borah P, Jindal J, Verma J (2000). Integrated management of bacterial leaf spot of mungbean with bacteriophages of *Xanthomonas axonopodis* pv. *Vignaeradiatae* and chemicals. J. Mycol. Plant Pathol.

[118] Lee CN, Lin JW, Chow TY, Tseng YH, Weng SF (2006). A novel lysozyme from *Xanthomonas oryzae* phage ɸXo411 active against *Xanthomonas* and *Stenotrophomonas*. Protein Expr Purif.

[119] Balogh B, Nga NTT, Jones JB (2018). Relative level of bacteriophage multiplication in vitro or in phyllosphere may not predict in planta efficacy for controlling bacterial leaf spot on tomato caused by *Xanthomonas perforans*. Front Microbiol.

[120] Dömötör D, Frank T, Rákhely G, Doffkay Z, Schneider G, Kovács T (2016). Comparative analysis of two bacteriophages of *Xanthomonas arboricola* pv. *Juglandis*. Infect. Genet. Evol..

[121] National Center for Biotechnology Information. Bethesda Maryland. 2021. https://www.ncbi.nlm.nih.gov/nuccore/LR743529. Accessed 8 Mar 2021.

[122] National Center for Biotechnology Information. Bethesda Maryland. 2021. https://www.ncbi.nlm.nih.gov/nuccore/LR743531. Accessed 8 Mar 2021.

[123] National Center for Biotechnology Information. Bethesda Maryland. 2021. https://www.ncbi.nlm.nih.gov/nuccore/LR743528. Accessed 8 Mar 2021.

[124] National Center for Biotechnology Information. Bethesda Maryland. 2021. https://www.ncbi.nlm.nih.gov/nuccore/MT210154. Accessed 8 Mar 2021.

[125] National Center for Biotechnology Information. Bethesda Maryland. 2021. https://www.ncbi.nlm.nih.gov/nuccore/MT119766. Accessed 8 Mar 2021.

[126] National Center for Biotechnology Information. Bethesda Maryland. 2021. https://www.ncbi.nlm.nih.gov/nuccore/AY986977. Accessed 8 Mar 2021.

[127] Inoue Y, Matsuura T, Ohara T, Azegami K (2006). Bacteriophage OP1, lytic for *Xanthomonas oryzae* pv. *Oryzae*, changes its host range by duplication and deletion of the small domain the deduced tail fiber gene. J. Gen. Plant Pathol.

[128] Yuzenkova J, Nechaev S, Berlin J, Rogulja D, Kuznedelov K, Inman R, Mushegian A, Severinov K (2003). Genome of *Xanthomonas oryzae* bacteriophage Xp10: an odd T-odd phage. J Mol Biol.

[129] National Center for Biotechnology Information. Bethesda Maryland. 2021. https://www.ncbi.nlm.nih.gov/nuccore/LR743530. Accessed 10 Mar 2021.

[130] National Center for Biotechnology Information. Bethesda Maryland. 2021. https://www.ncbi.nlm.nih.gov/nuccore/LR743532. Accessed 10 Mar 2021.

[131] National Center for Biotechnology Information. Bethesda Maryland. 2021. https://www.ncbi.nlm.nih.gov/nuccore/MN364664. Accessed 20 May 2021.

[132] Inoue Y, Matsuura T, Ohara T, Azegami K (2006). Sequence analysis of the genome of OP2, a lytic bacteriophage of *Xanthomonas oryzae* pv. *Oryzae*. J Gen Plant Pathol.

[133] National Center for Biotechnology Information. Bethesda Maryland. 2021. https://www.ncbi.nlm.nih.gov/nuccore/JN022534. Accessed 10 Mar 2021.

[134] National Center for Biotechnology Information. Bethesda Maryland. 2021. https://www.ncbi.nlm.nih.gov/nuccore/MH059633. Accessed 10 Mar 2021.

[135] Ghei OK, Eisenstark A, Consigli RA, To CM (1968). Structure and composition of *Xanthomonas pruni* bacteriophage. J. Gen. Virol..

[136] National Center for Biotechnology Information. Bethesda Maryland. 2021. https://www.ncbi.nlm.nih.gov/nuccore/MH206183. Accessed 10 Mar 2021.

[137] National Center for Biotechnology Information. Bethesda Maryland. 2021. https://www.ncbi.nlm.nih.gov/nuccore/MH218848. Accessed 10 Mar 2021.

[138] National Center for Biotechnology Information. Bethesda Maryland. 2021. https://www.ncbi.nlm.nih.gov/nuccore/MH206184. Accessed 10 Mar 2021.

[139] National Center for Biotechnology Information. Bethesda Maryland. 2021. https://www.ncbi.nlm.nih.gov/nuccore/MN263053. Accessed 10 Mar 2021.

[140] Lin NT, You BY, Huang CY, Kuo CW, Wen FS, Yang JS, Tseng YH (1994). Characterization of two novel filamentous phages of *Xanthomonas*. J. Gen. Virol..

[141] Tseng YH, Lo MC, Lin KC, Pan CC, Chang RY (1990). Characterization of filamentous bacteriophage ΦLf from *Xanthomonas campestris* pv. *Campestris*. J. Gen. Virol..

